# Improving the Convergence Rate in Affine Registration of PET and SPECT Brain Images Using Histogram Equalization

**DOI:** 10.1155/2013/760903

**Published:** 2013-05-12

**Authors:** D. Salas-Gonzalez, J. M. Górriz, J. Ramírez, P. Padilla, I. A. Illán

**Affiliations:** Department of Signal Theory Networking and Communication, University of Granada, ETSIIT, 18071 Granada, Spain

## Abstract

A procedure to improve the convergence rate for affine registration methods of medical brain images when the images differ greatly from the template is presented. The methodology is based on a histogram matching of the source images with respect to the reference brain template before proceeding with the affine registration. The preprocessed source brain images are spatially normalized to a template using a general affine model with 12 parameters. A sum of squared differences between the source images and the template is considered as objective function, and a Gauss-Newton optimization algorithm is used to find the minimum of the cost function. Using histogram equalization as a preprocessing step improves the convergence rate in the affine registration algorithm of brain images as we show in this work using SPECT and PET brain images.

## 1. Introduction

 Emission computed tomography (ECT) has been widely employed in biomedical research and clinical medicine during the last three decades. ECT differs fundamentally from many other medical imaging modalities in that it produces a mapping of physiological functions as opposed to imaging anatomical structure. Tomographic radiopharmaceutical imaging, or ECT, provides in vivo three-dimensional maps of a pharmaceutical labeled with a gamma ray emitting radionuclide. The distribution of radionuclide concentrations isestimated from a set of projectional images acquired at many different angles around the patient. In this work, two different image modalities will be used: positron emission tomography (PET) and single photon emission computed tomography (SPECT).

Positron emission tomography (PET) is noninvasive, nuclear medicine imaging technique which produces a three-dimensional image of functional processes in the body. The system detects pairs of gamma rays emitted indirectly by a positron-emitting radionuclide (tracer), which is introduced into the body on a biologically active molecule. When the tracer is ^18^F-Fluorodeoxyglucose (F-FDG), its concentration gives us information about tissue metabolic activity, measuring the brain's rate of glucose metabolism. Images of tracer concentration in 3-dimensional space within the brain are then reconstructed by computer analysis.

On the other hand, SPECT is a widely used technique to study the functional properties of the brain. SPECT brain imaging techniques employ radioisotopes which decay emitting predominantly a single gamma photon. When the nucleus of a radioisotope disintegrates, a gamma photon is emitted with a random direction which is uniformly distributed in the sphere surrounding the nucleus. If the photon is unimpeded by a collision with electrons or other particles within the body, its trajectory will be a straight line or “ray.” In order for a photon detector external to the patient to discriminate the direction that a ray came from, a physical collimation is required. Typically, lead collimator plates are placed prior to the detector's crystal in such a manner that the photons coming from all but a single direction are blocked by the plates. This guarantees that only photons coming from the desired direction will strike the photon detector.

After image acquisition, when filtering and reconstruction are done, some additional preprocessing steps are needed before using functional brain images for computer aided diagnosis systems. The differences between brains of different subjects require the normalization of the images with respect to a reference template [1]. Image normalization allows to perform voxel-to-voxel comparison between same regions of different images [2, 3]. The general affine model with 12 parameters is usually chosen as a first normalization procedure before to proceed with a more complex nonrigid spatial transformation model [4–7]. Affine registration of tomography brain images is a very important task in biomedical image analysis [8–10].

The methods and approaches presented in this work have been motivated in the context of fusion of PET/MRI images. While the authors were developing a software for fusion of brain images, they were working under some hypothesis to render practical the requirements this software would then be used for: (i) using only affine registration because other nonlinear registration methods could possibly produce undesirable warping effects due to the intrasubject, intermodality nature of the registration problem studied in this work; (ii) providing reliability and robustness and increasing the convergence rate of the algorithms as far as possible; (iii) using the same anatomical brain image as a reference template for each image. This latter point was motivated by practical purposes as the registration method proposed here was developed in the context of a real application of brain image fusion of PET/MRI. Furthermore, the proposed methodology has been also tested in fusion of SPECT/MRI images. Therefore, the goal is to register the functional brain images (PET or SPECT) to the anatomical Magnetic Resonance Imaging (MRI). 

In this work, a histogram equalization of the original tomography brain image is performed. We enhance the contrast of images by transforming the intensity values in the image, so that the histogram of the output image approximately matches the histogram of the reference template. Experimental results using positron emission tomography and single photon emission computed tomography brain images show that the preprocessing of these images using histogram equalization improves the convergence rate of the affine registration algorithm.

This paper is organised as follows.  Section 2 introduces the SPECT and PET database used in this work; it also states the image registration problem expressed by a matrix multiplication and the Gauss-Newton optimization algorithm used to estimate the affine parameters. This section also presents the preprocessing step of the intensity values using histogram matching. In Section 3, the results are discussed. Lastly, in Section 4, the conclusions are given.

## 2. Materials and Methods

### 2.1. SPECT Database

 The SPECT database is from a current study of the Alzheimer's disease performed by the “Virgen de las Nieves” Hospital in Granada (Spain). In this work, we choose 50 images labelled by experts as Normal Controls, although the results presented here do not change if brain images labelled as late Alzheimer's disease subjects are chosen.

The patients were injected with a gamma emitting ^99m^Tc-ECD radiopharmaceutical, and the SPECT raw data was acquired by a three-head gamma camera. A total of 180 projections were taken for each patient with a 2-degree angular resolution. The images of the brain cross-sections were reconstructed from the projection data using the filtered backprojection (FBP) algorithm in combination with a Butterworth noise removal filter [11–14].

### 2.2. PET Database

 PET images used in the preparation of this work were obtained from the Alzheimer's Disease Neuroimaging Initiative (ADNI) database (http://www.loni.ucla.edu/ADNI). The ADNI was launched in 2003 by the National Institute on Aging (NIA), the National Institute of Biomedical Imaging and Bioengineering (NIBIB), the Food and Drug Administration (FDA), private pharmaceutical companies, and nonprofit organizations, as a 60-million, 5-year public-private partnership. The primary goal of ADNI has been to test whether serial magnetic resonance imaging (MRI), positron emission tomography (PET), other biological markers, and clinical and neuropsychological assessment can be combined to measure the progression of mild cognitive impairment (MCI) and early Alzheimer's disease (AD). Determination of sensitive and specific markers of very early AD progression is intended to aid researchers and clinicians to develop new treatments and monitor their effectiveness, as well as lessen the time and cost of clinical trials.

The Principle Investigator of this initiative is Michael W. Weiner, MD degree, VA Medical Center and University of California, San Francisco. ADNI is the result of efforts of many coinvestigators from a broad range of academic institutions and private corporations, and subjects have been recruited from over 50 sites across the US and Canada. The initial goal of ADNI was to recruit 800 adults, ages 55 to 90, to participate in the research—approximately 200 cognitively normal older individuals to be followed for 3 years, 400 people with MCI to be followed for 3 years, and 200 people with early AD to be followed for 2 years.

FDG-PET scans were acquired according to a standardized protocol. A 30-minute dynamic emission scan, consisting of 6 5-minute frames, was acquired starting from 30 minutes and after the intravenous injection of 5.0–0.5 mCi of ^18^F-FDG, as the subjects, who were instructed to fast for at least 4 h prior to the scan and lay quietly in a dimly lit room with their eyes open and minimal sensory stimulation. Data were corrected for radiation-attenuation and scatter using transmission scans from Ge-68 rotating rod sources and reconstructed using measured attenuation correction and image reconstruction algorithms specified for each scanner (http://www.loni.ucla.edu/ADNI/Data/ADNI_Data.shtml). Following the scan, each image was reviewed for possible artifacts at the University of Michigan, and all raw and processed study data was archived.

### 2.3. Brain Affine Registration

An affine transformation maps from position **x** in one image to **y** in another via matrix **M**, where the 12 matrix *m*_*ij*_ are the unknown parameters. For each voxel **x** = (*x*_1_, *x*_2_, *x*_3_) in an image, the affine transformation into the coordinates **y** = (*y*_1_, *y*_2_, *y*_3_) is expressed by a matrix multiplication **y** = **M** **x**, where

(1) $$\begin{array}{c} \left(\begin{array}{c} y_{1}\\ y_{2}\\ y_{3}\\ 1 \end{array}\right)=\left(\begin{array}{cccc} m_{11} & m_{12} & m_{13} & m_{14}\\ m_{21} & m_{22} & m_{23} & m_{24}\\ m_{31} & m_{32} & m_{33} & m_{34}\\ 0 & 0 & 0 & 1 \end{array}\right)\left(\begin{array}{c} x_{1}\\ x_{2}\\ x_{3}\\ 1 \end{array}\right). \end{array}$$

The goal of the affine image registration is to find the 12 components **m** of the matrix **M** describing a transformation that best matches both images (the source and the template) together.

**Figure 1:**
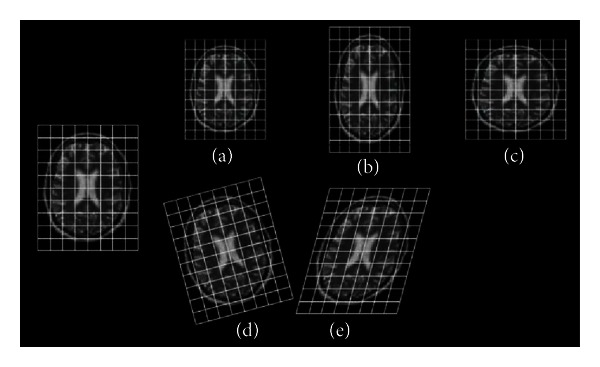
Left image: original MRI brain transaxial image. (a) Balanced diagonal elements result in global rescaling. (b) Unbalanced diagonal elements produce anisotropic rescaling along the *y*-axis. (c) Unbalanced diagonal elements produce anisotropic rescaling along the *x*-axis. (d) Specific balanced relations between off-diagonal elements in the matrix produce rotations. (e) An unbalance change in the off-diagonal elements resulting in shears along one of the two axes.

The affine transformation is parametrized by 12 parameters. *m*_11_, *m*_22_, *m*_33_ model zooms of the original image. These parameters allow us to scale the medical image. For example, if *m*_11_ > 1, the transformed image is greater than the original one in the *x*-axis. A zoom in the image is desirable when the image and template do not have the same size. Translations in *x*-, *y*-, or *z*-axis are parametrized by the component *m*_14_, *m*_24_, or *m*_34_, respectively, and provide a manner to center the image and the template. Shears are modeled by the nondiagonal elements of the matrix (*m*_12_, *m*_13_, *m*_23_, *m*_21_, *m*_31_, and *m*_32_).  Figure 1 shows the effects produced by different elementary 2-D affine transformations in a transaxial 2D slice MRI image. Left image is a random 2-D transaxial slice. Figures 1(a), 1(b), and 1(c) show the effects produced by changes in the diagonal elements in the 2-D affine matrix: balanced diagonal elements result in global rescaling (Figure 1(a)); unbalanced diagonal elements produce anisotropic rescaling along the *y*-axis (Figure 1(b)) or the *x*-axis (Figure 1(c)). Some specific relations between off-diagonal elements in the matrix produce rotations (Figure 1(d)), and, lastly, Figure 1(e) depicts the effects of an unbalance change in the off-diagonal elements resulting in shears along one of the two axes. 

Before the application of an affine transformation to the original functional image, it is convenient to smooth the source image to improve accuracy [15]. This step decreases the number of potential local minima in the optimization task. On the other hand, the intensity values in the original image are referred to the center of the voxels. After the application of an affine transformation to an image, the centers of the voxels of the image rarely are placed in the centers of the voxels in the transformed image. Therefore, interpolation is needed in order to estimate the intensity value in the center of the voxels for the transformed image [16, 17].

### 2.4. Gauss-Newton Optimization Algorithm

The parameters **m** can be estimated by minimizing given cost function. The cost function (cf) is chosen as the mean squared difference between the images as follows:

(2) $$\begin{array}{c} ᴄꜰ=\sum_{i}^{}b_{i}{\left(m\right)}^{2}=\sum_{i}^{}{\left(f\left(Mx_{i}\right)-g\left(x_{i}\right)\right)}^{2}, \end{array}$$

where *f* denotes the source image and *g* the template. In this work, the Gauss-Newton algorithm is used to estimate the matrix components in **M** by finding the minimum of the cost function cf.

The Gauss-Newton algorithm (GN) can be viewed as a modification of Newton's method with line search [18]. It is an iterative procedure which allows to find the minimum of a sum of squares. Starting with an initial guess **m** ^0^, at each iteration, the value of **m** is updated using the following rule:

(3) $$\begin{array}{c} m^{t+1}=m^{t}+\delta \end{array}$$

with **δ** satisfying the equation

(4) $$\begin{array}{c} \left(J^{T}J\right)\delta =-J^{T}b, \end{array}$$

where **b** is the vector of functions *b*_*i*_ and **J** is the Jacobian matrix of **b** with respect to **m** evaluated at **m** ^*t*^. This optimization method is like Newton's method with line search, but in that case, the Hessian is approximated using

(5) $$\begin{array}{c} \nabla^{2}f_{k}\approx J^{T}J. \end{array}$$

This choice avoids the computation of the individual Hessians of the residuals which sometimes can be difficult to compute.

### 2.5. Histogram Equalization

Histogram matching is a procedure where a series of histogram-equalization steps is used to obtain an image with a histogram, that is, close to a prespecified histogram.

Suppose that the desired or specified normalized histogram is *p*_*d*_(*t*), with the desired image being represented as *d*, having the normalized gray levels *t* = 0,1, 2,…, *L* − 1. Now, the given image *f* with the PDF *p*_*f*_(*r*) may be histogram-equalized by the transformation

(6) $$\begin{array}{c} C_{1}\left(r\right)=\int_{0}^{r}p_{f}\left(w\right)dw;\;0\leq r\leq 1. \end{array}$$

We may also derive a histogram-equalizing transform for the desired (but as yet unavailable) image as

(7) $$\begin{array}{c} C_{2}\left(t\right)=\int_{0}^{t}p_{d}\left(w\right)dw;\;0\leq t\leq 1. \end{array}$$

Observe that, in order to derive a histogram-equalizing transform, we need only the PDF of the image; the image itself is not needed.

When you supply a desired histogram, mathematically histogram equalization consists of choosing the grayscale transformation **T** to minimize

(8) $$\begin{array}{c} ||C_{1}\left(T\left(t\right)\right)-C_{2}\left(t\right)||, \end{array}$$

where *C*_2_ is the cumulative histogram of the reference image (the desired image represented as *d*), and *C*_1_ is the cumulative sum of the image *f* for all intensities *t*. This minimization is subject to the constraints that **T** must be monotonic and *C*_1_(**T**(*a*)) cannot overshoot *C*_2_(*a*) by more than half the distance between the histogram counts at a given intensity value *a*. Then, the transformation **T** will be used to map the gray levels in the image *f* (or the colormap) to their new values.

**Figure 2:**
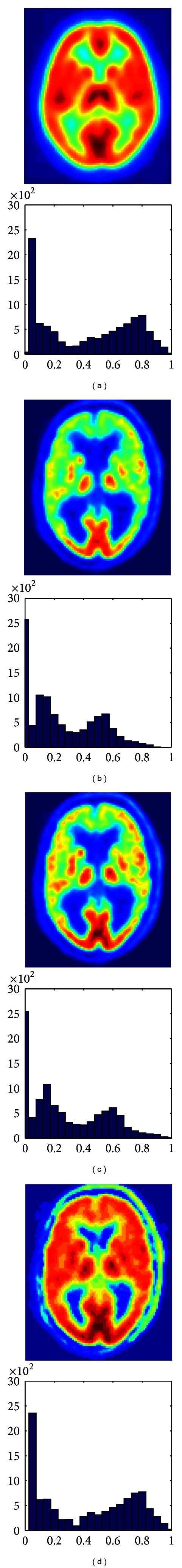
(a) Reference PET template. (b) Source PET brain image. (c) Source image normalized to the greater value of reference PET template. (d) Enhanced image using histogram matching.

Figure 2 depicts four different brain PET images and their histograms.  Figure 2(a) is a reference PET image.  Figure 2(b) shows a random transaxial 2-D PET image as it is obtained after reconstruction.  Figure 2(c) shows a version of the same source PET image but with a dynamic range expansion to the same interval as the reference image ([01]).  Figure 2(d) shows the source image after the application of a histogram matching procedure using the histogram of the reference image in Figure 2(a) as a prespecified histogram. Let us see that in that case, both images (in Figures 2(a) and 2(d)) and their histograms are very similar.

### 2.6. Summary

 The procedure we follow to preprocess the functional brain images before to proceed with an affine registration to a template is summarized in this subsection.

- Firstly, we apply a mask in the source images and we consider only those voxels with intensity values greater than a given threshold. This step is done to discard those voxels in the image outside the brain.
- Secondly, we calculate the histogram of the template image.
- Then, we perform histogram matching and we adjust the intensity values in the source images to the intensity of the reference template.
- Lastly, we build a cost function (using the mean squared difference between source image and reference template) and we estimate the 12 parameters of the affine matrix using a Gauss-Newton optimization procedure.

## 3. Results and Discussion

The proposed methodology has been tested for 50 different SPECT and PET images. The images have been spatially normalized to a common template using a general affine model with 12 parameters.

**Figure 3:**
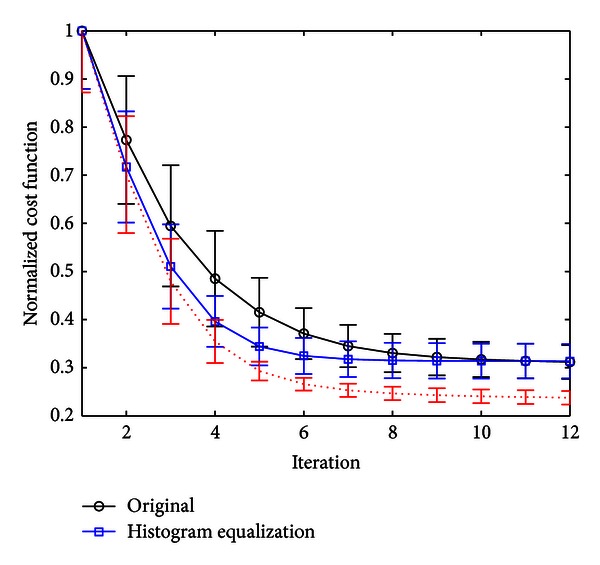
Circles: mean normalised cost function value of 50 images versus number of iterations for the original PET brain images with intensity values expanded from 0 to 1. Rectangles: mean normalised cost function calculated applying the affine matrix calculated in each iteration using the preprocessed PET images to the original brain PET images. Dotted red line: mean normalised cost function value of 50 images versus number of iterations for preprocessed images using histogram matching.

In order to test the performance of the proposed method, we estimate the 12 components of the affine matrix after adjustment of the intensity of the images using histogram matching. We also estimate the general affine transformation matrix for the original images with intensity values expanded from 0 to 1, which is the same range as the template image. Therefore, this dataset is linearly normalized in intensity to the maximum. We compare the results for these two datasets by means of a plot of the mean normalized cost function versus the number of iterations. The mean normalized cost function for the transformed images is plotted in red using dots in Figures 3 and 5. Let us note that this result cannot be compared directly with the performance of the affine registration optimization method using the original images as the cost function (2) is intensity dependent. In order to perform a fair comparison, the affine matrix calculated in each iteration using the enhanced images is applied to the original brain image. Then, the cost function is calculated and plotted in blue squares (labelled as “histogram equalization”). The latter can be directly compared to the values of the normalized cost function in each iteration when the original images are used (labelled as “original” and plotted with black circles in Figures 3 and 5.

Figure 3 shows the mean normalised cost function value versus the number of iterations when the proposed methodology was tested using the PET database. The error bars are calculated using the standard deviation. In that case, preprocessing the images using histogram matching allows to improve the convergence rate of the algorithm. Specifically, images preprocessed using histogram equalization reach in the sixth iteration the same mean normalized value of the cost function as the original images in the ninth iteration which means a significant reduction in the convergence rate.

**Figure 4:**
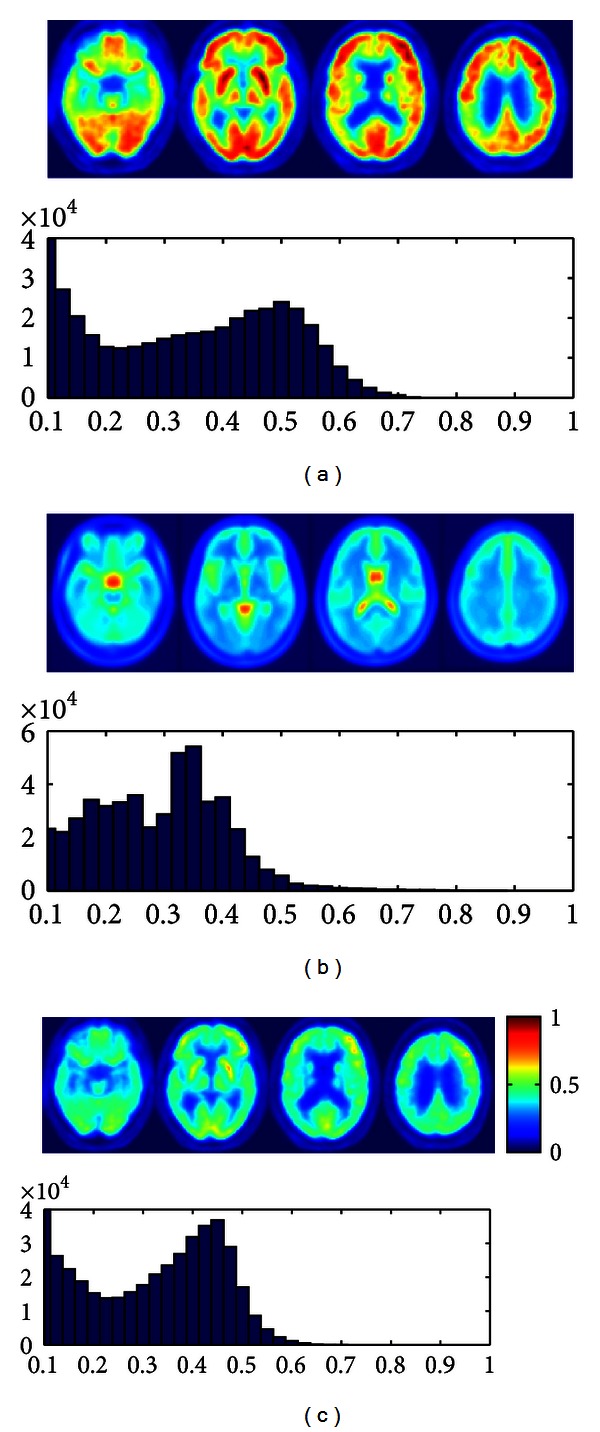
(a) Four transaxial slices of mean of 50 original PET images and its histogram of intensity values. (b) Four transaxial cuts of the reference template and the histogram of intensity values. (c) Four transaxial slices of mean of 50 preprocessed PET images using histogram equalization.

Figure 4(a) shows four transaxial cuts of the mean PET image after affine normalization used in this work and its corresponding histogram of intensity values.  Figure 4(b) shows the reference template which is used in this work, and Figure 4(c) depicts four transaxial cuts of the mean PET image after affine normalization for the 50 PET images which were preprocessed using histogram matching. In that case, both the transaxial images and the histogram show that the intensity values of the images are similar to the intensity values of the image template in Figure 4(b).

**Figure 5:**
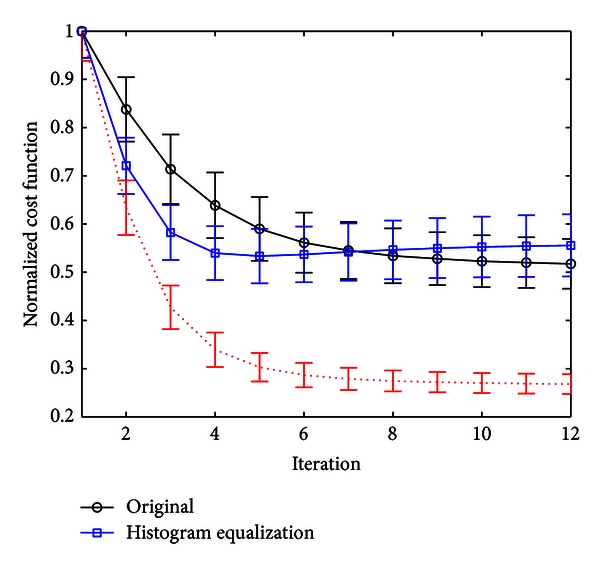
Circles: mean normalised cost function value of 50 images versus number of iterations for the original SPECT brain images with intensity values expanded from 0 to 1. Rectangles: mean normalised cost function calculated applying the affine matrix calculated in each iteration using the preprocessed SPECT images to the original brain SPECT images. Dotted red line: mean normalised cost function value of 50 images versus number of iterations for preprocessed images using histogram matching.

Figure 5 shows the normalized mean cost function versus the number of iterations for 50 original SPECT brain images (denoted by circles) and the same images preprocessed using a histogram matching procedure to the reference template (with squares). The error bars are calculated using the standard deviation. Analogously to the previous case, when PET images were considered, this plot has been calculated applying the affine matrix estimated in each iteration using the preprocessed images to the original brain SPECT images. Furthermore, as in Figure 3, the red dotted line is the mean normalised cost function for the preprocessed images using histogram matching. The figure shows that preprocessing the images leads to an improvement of the convergence rate. Specifically, the algorithm reaches in only four iterations a normalised cost function value very similar to the value which is obtained using the original SPECT images in the eighth iteration. When the number of iterations is greater than 8, enhanced images obtain a mean value of the normalized cost function slightly greater than using the original images. Nevertheless, the proposed method is also useful for practical purposes in that case. For instance, histogram adjustment of original images can be used to estimate the affine transformation matrix up to the fourth iteration, and then this estimate can be used as initial guess **m** ^0^ in the Gauss-Newton optimization algorithm to calculate the 12 affine parameters using the original images.

**Figure 6:**
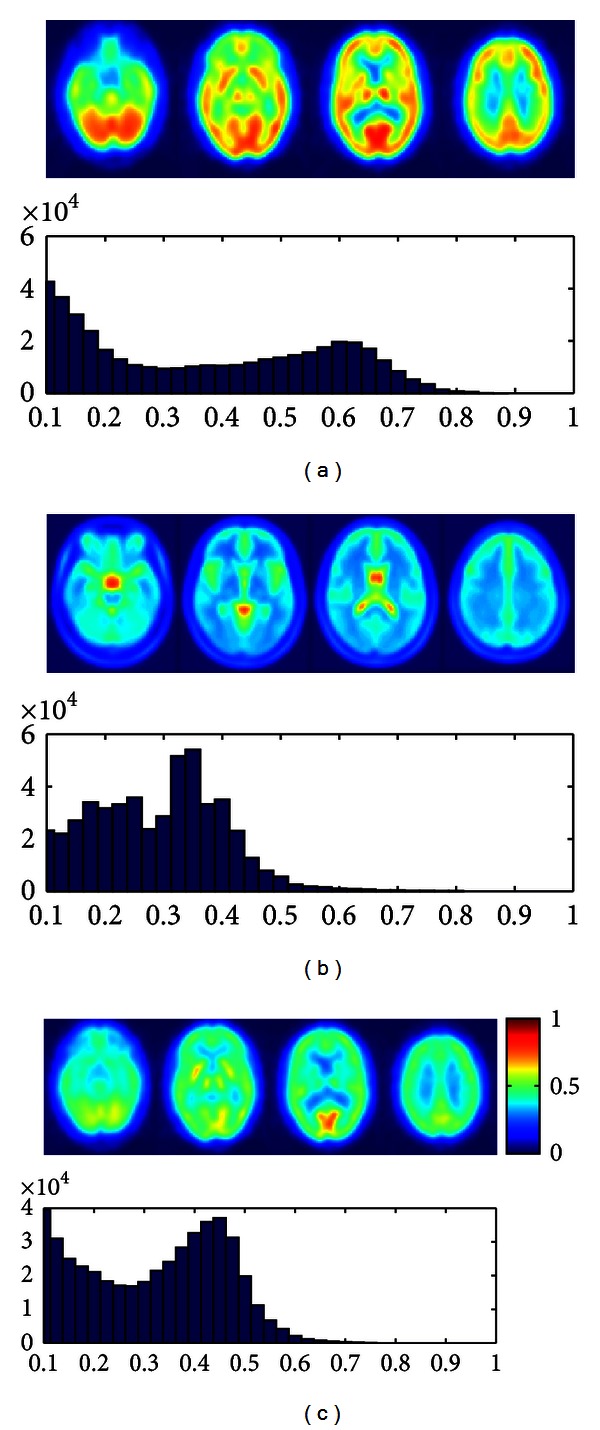
(a) Four transaxial slices of mean of 50 original SPECT images and its histogram of intensity values. (b) Four transaxial cuts of the reference template and the histogram of intensity values. (c) Four transaxial slices of mean of 50 preprocessed SPECT images using histogram matching.

Figure 6(a) depicts four transaxial brain images showing the mean after affine registration of 50 SPECT images considered in this work. The same is shown for the reference template, and let us note that Figures 6(b) and 4(b) are the same, as the same reference brain template image was used for the affine registration of the SPECT and PET images.  Figure 6(c) depicts four transaxial brain cuts with transformed intensity values using histogram matching. Analogously to the case in which PET images were considered, Figure 6(c) shows that after histogram transformation, both the images and the histogram of intensity values are quite similar to the template in Figure 6(b).

 In this work, histogram matching is used as a useful tool which is able to increase the convergence rate of affine transformation methods widely used in the literature. Histogram equalization is a common procedure which is usually used to enhance contrast in image processing. We have shown that this approach can be used in the context of affine registration of brain images, specifically when the intensity values of the reference template differ from the source image which is the case in intermodality image fusion of brain images. Specifically, in this work, the convergence rate of the affine registration using Gauss-Newton optimization algorithm has been compared using 50 PET and 50 SPECT images. When the images were preprocessed using histogram matching (using the histogram of the template image as prespecified desired histogram), the convergence rate of the algorithm has been improved. 

## 4. Conclusion

 In this work, a procedure to improve the convergence rate in the context of affine registration of tomography brain images has been presented. The proposed methodology is based on a histogram matching; the intensity values in the original image are preadjusted using the information of the histogram of the reference template. This intensity normalization has been tested on different imaging modalities: single photon emission computed tomography and positron emission tomography. This approach has been shown to improve the convergence rate of the affine registration of tomography brain images specially when there is a great difference between intensity values of the reference template and the images.
